# Sero-prevalence and spatial distribution of Rift Valley fever infection among agro-pastoral and pastoral communities during Interepidemic period in the Serengeti ecosystem, northern Tanzania

**DOI:** 10.1186/s12879-018-3183-9

**Published:** 2018-06-14

**Authors:** Abade Ahmed, Jabir Makame, Fyumagwa Robert, Keyyu Julius, Matee Mecky

**Affiliations:** 1 Tanzania Field Epidemiology and Laboratory Training Program, Ministry of Health, Community Development, Gender, Elderly and Children, P.O Box 71286, Ocean Road, Dar es Salaam, Tanzania; 2 Department of Microbiology and Immunology, Muhimbili University of Health and Allied Science, Dar es Salaam, Tanzania; 3 Tanzania Wildlife Research Institute, Arusha, Tanzania

**Keywords:** Rift valley, Serengeti ecosystem, Inter-epidemic human, Tanzania

## Abstract

**Background:**

In the past two decades, Rift Valley Fever (RVF) outbreaks have been reported twice in Tanzania, with the most recent outbreak occurring in 2006/07. Given the ecology and climatic factors that support mosquito vectors in the Serengeti ecosystem, we hypothesized a continued transmission of RVF virus (RVFV) during interepidemic periods. This study was carried out to determine sero-prevalence, spatial distribution and factors associated with RVF in at-risk agro-pastoral and pastoral communities in the Serengeti Ecosystem in northern Tanzania.

**Methods:**

A cross sectional study was carried out to establish the general exposure to RVFV by detecting anti–RVFV IgG and anti–RVFV IgM using ELISA techniques. The health facilities where human subjects were blood sampled concurrent with interviews included Bunda District Designated Hospital, Wasso DDH, Endulen hospital, Arash, Malambo, Olbabal, and Piyaya dispenaries (Ngorongoro district) and Nyerere DDH (Serengeti district) respectively. In addition, human subjects from Lamadi ward (Busega district) were recruited while receiving medical service at Bunda DDH. We conducted logistic regression to assess independent risk factor and mapped the hotspot areas for exposure to RVFV.

**Results:**

A total of 751 subjects (males = 41.5%; females = 58.5%) with a median age of 35.5 years were enrolled at out-patient clinics. Of them, 34 (4.5, 95%CI 3.3–6.3%) tested positive for anti–RVFV IgG. Of the 34 that tested positive for anti–RVFV IgG, six (17.6%) tested positive for anti–RVFV IgM. Odds of exposure were higher among pastoral communities (aOR 2.9, 95% C.I: 1.21–6.89, *p* < 0.01), and agro-pastoral communities residing in Ngorongoro District (aOR 1.8, 95% C.I 1.14–3.39, *p* = 0.03). Hotspot areas for exposure to RVFV were Malambo, Olbalbal and Piyaya wards in Ngorongoro district, and Lamadi ward in Busega district.

**Conclusions:**

The study found both previous and recent exposure of RVFV in humans residing in the Serengeti ecosystem as antibodies against both IgG and IgM were detected. Detection of anti-RVF IgM suggests an ongoing transmission of RVFV in humans during inter-epidemic periods. Residents of Ngorongoro district were most exposed to RVFV compared to Bunda and Serengeti districts. Therefore, the risk of exposure to RVFV was higher among pastoral communities compared to farming communities.

## Background

Rift Valley Fever (RVF) is a viral zoonotic fever caused by RVF virus (RVFV), a member of the genus Phlebovirus in the family Bunyaviridae [1–3]. It is an illness characterized by deaths and abortion storms primarily in goats, sheep, and cattle [4]. The disease also affects humans, dogs, camels and wildlife [5]. Wild animals such as African buffalo (*Syncerus caffer*), black rhino (*Diceros bicornis*), lesser kudu (*Tragelaphus imberbis*), impala (*Aepyceros melampus*), kongoni (*Alcelaphus buselaphus*), monkeys (Cercopithecus spp.), waterbuck (*Kobus ellipsiprymnus*) and African elephants (*Loxodonta africana*) have shown to be exposed to the virus [6, 7].

Tanzania has experienced 10 epidemic episodes of RVF since 1930s. During the last RVF epidemic a case fatality of 46% was reported among humans [6–8]. Death of animals due to the disease was estimated to cost around 6 million US dollars, with external market flow dropping by 54% [8] Shortage of meat and milk probably caused acute malnutrition [9]. Most of the markets were closed and cost of alternative source of protein such as fish and chicken was very high [9, 10]. In humans, RVFV usually causes mild fever that is often associated with spontaneous recovery [6, 9]. However, infections characterized by flu-like illness, headache, muscles and joint pain, diarrhoea, vomiting [11] anorexia and high respiratory rate [12] do occur. RVFV may cause serious hepatitis and liver necrosis [6, 7] as well as other complications including loss of eyesight, meningo-encephalitis and haemorrhagic fever [6, 13]. Economically, apart from high livestock loss, the epidemic may affect the tourism industry that contributes over 17% of GDP in Tanzania [14] because of fear among tourists of contacting the disease from affected wildlife and loss of animal species that are sought for game viewing.

Despite the severity of the RVF, little is known regarding prevalence and exposure status of humans in different ecosystems in Tanzania. Previous studies conducted in Kilombero valley in eastern Tanzania showed high transmission of the disease in livestock, with sero-prevalence of 5.5% among animals born after last epidemic of 2007 [9]. Correspondingly, current study in the Serengeti ecosystem, which is the sister study to this, has found recent exposure among domestic animals and wildlife. Cattle and sheep recorded IgM prevalence of 5.7% while buffaloes recorded 3.1% of IgM prevalence (Nyarobi unpublished). RVFV-RNA was extracted from 2.7% of mosquitoes pools studied in the ecosystem (Nyarobi unpublished). This has raised need to understand the current disease burden in humans and predict the possibility of future outbreak. Therefore, this study was done to determine the exposure status of RVFV, its spatial distribution and factors associated with exposure to RVFV among pastoral and agro-pastoral communities of the Serengeti ecosystem.

## Methods

### Study area and population

**Fig. 1:**
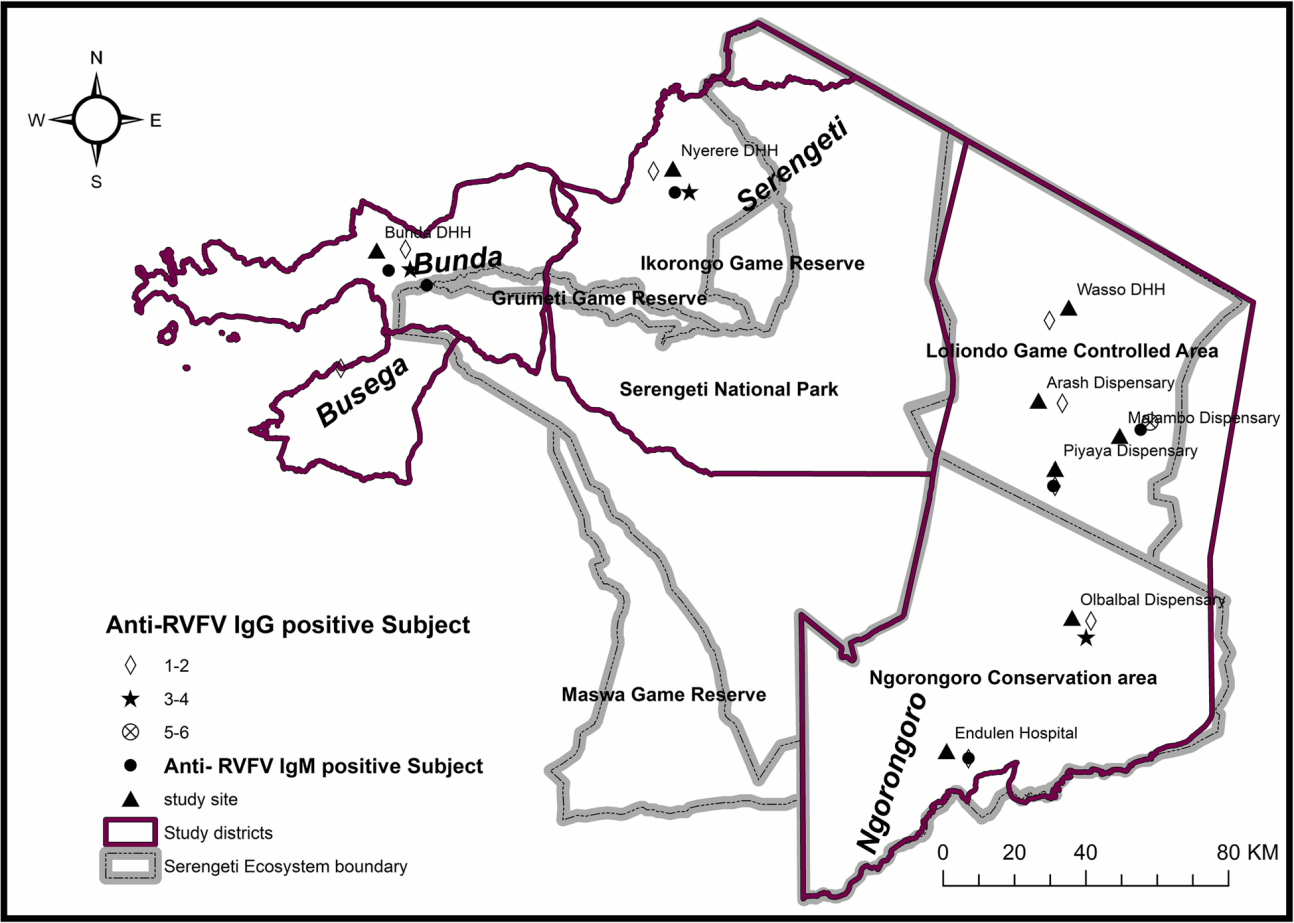
Map of Serengeti ecosystem showing districts where anti RVF IgG seropositive humans were identified (see attached document)

The study was conducted in the Serengeti ecosystem in northern regions of Tanzania. The area included three districts of Bunda, Ngorongoro and Serengeti in the Serengeti ecosystem, (Fig. 1). However, some human subjects were enrolled from Lamadi ward in Busega district. The area is located along the border with Kenya, close to the equator, between 2o to 4o *S. minimum* temperature ranges between 15-21oC, while maximum ranges between 24-27oC. The rainfall is highly seasonal with peaks in March to May and November to December. Mean annual rainfall in the Serengeti varies from 1050 mm in the northwest to 550 mm in the southeast. The area is covered with rich volcanic soil that supports the growth of vegetation. Meshwork of streams of water bodies are found throughout the area. The ecosystem extends to the shores of Lake Victoria in the west, (where Bunda and Busega districts are located), Lake Eyasi in the south and the Great Rift Valley to the east, covering Ngorongoro Conservation Area (NCA) and Loliondo Game Controlled Area (LGCA) in Ngorongoro district. The study also extended to the western side in Serengeti and Bunda districts (Fig. 1). Soil type, grassy plain, temperature and rainfall of the ecosystem favor the endemicity of the disease in the area. The communities surrounding the area are mainly of pastoral and agro-pastoral communities. Pastoral communities are those deriving their income solely by keeping animals like cattle, sheep, goats, donkey and dogs. Agro-pastoral communities are those depending on crop cultivation supplemented with animal keeping mostly in small scale.

### Study design and setting

A cross-sectional, health facility based study was conducted on humans in the Serengeti ecosystem during the dry season of August–October, 2014. The health facilities where the study were conducted included Wasso District Designated Hospital (DDH), Endulen hospital, Malambo, Piyaya, Arash and Olbabal dispensaries in Ngorongoro District; Bunda DDH in Bunda district and Nyerere DDH in Serengeti district respectively. As a hospital-based study, participants from Lamadi ward (Busega district) bordering Bunda district were also enrolled as they were frequently seeking medical services at Bunda DDH. We enrolled patients aged 5 years and above attending outpatient department (OPD) regardless of their clinical presentation. Patients who could not give assent or consent and those who were critically ill to give information were excluded from the study. A total of 751 human subjects were included in the study. Probability proportion to population size was used to allocate the calculated sample size to the three districts’ health facilities. The three districts of Ngorongoro, Serengeti and Bunda districts were allocated 210, 241 and 300 study respondents respectively. Based on geographical nature and sparse population distribution of Ngorongoro district, apart from district hospital, more health facilities were included in the study to allow wider coverage of all parts of the district as much as possible. The additional health facilities were Endulen hospital, as well as Malambo, Piyaya, Arash, and Olbalbal dispensaries. These are the main health facilities in the area that provide services to all population in the study area. Simple random sampling technique was used to select participants among out-patients who visited the respective health facility for medical services or individuals who escorted relatives for medical services in the eight health facilities in the three district i.e. six health facilities in Ngorongoro district, one in Bunda and one in Serengeti districts respectively. The coordinates of the study sites were recorded using a Global Positioning System (GPS) device for subsequent use in marking the sampling sites and indicate locations with sero-positive human subjects in the study area. In Tanzania, the mean straight-line distance to a health facility is about 4.2 km (SD 3.9 km) [15].

### Laboratory investigation

Approximately, 4 mls of whole human blood was drawn aseptically from each patient recruited in the study. Approximately 1–2 mls of sera was obtained from each blood sample through centrifugation, and transferred into screw capped cryovials and stored at -20 °C freezer awaiting serological analysis. Sera from patients were tested for the presence of anti-RVFV IgG antibody using competitive Enzyme linked Immuno-sorbent Assay (ID.Vet Innovative Diagnostics, Grabels, France). The test has excellent sensitivity and specificity of 100%. Detection of anti-RVFV IgM was done using capture ELISA kit, to all anti-RVFV IgG positive samples as described by the manufacturer (Biological Diagnostics Supply Limited -BDSL, UK). The sensitivity test was 100% and specificity ranged from 97.4 to 99.4% [16]. Validation of the results was done by repeating all positive and 10% of the negative samples and found 100% concordance between the first test and observed validation test.

### Data management and statistical analysis

Information on social demographic characteristics including age, sex, level of education, occupation and area of residence was collected. Median, range, and proportion of seropositive samples among those tested for anti-RVFV IgG (general exposure status) and anti-RVFV IgM (recent exposure status) were calculated. A number of socio-demographic factors associated with RVFV sero-positivity were assessed using prevalence Odds Ratio (OR) as a measure of association at 95% confidence interval. Socio-demographic factors with *p*-value ≤0.05 were considered as statistically significant in bivariate analysis. All variables with *p* ≤ 0.2 at bivariate level were entered into multivariate logistic regression model. Variables with p-value ≤0.05 at multivariate level were considered independently associated with RVF seropositivity.

## Results

A total of 751 participants were enrolled in this study. Their median age was 35.5 years (range = 5–90 years). Of them, 439 (58.5%) were females, 294 (45.9%) were small scale farmers, 420 (63.8%) had primary education and 300 (40%) were from Bunda district (Table 1).

**Table 1:** Socio-demographic distribution of study respondents in the Serengeti ecosystem, 2014

	District			Enrolled
Variable	Bunda *N* = 300 (%)	Ngorongoro *N* = 210 (%)	Serengeti *N* = 241(%)	Total 751 (%)
Age group				
< 15	2 (1)	16 (8)	24 (11)	42 (5.6)
15–29	67 (22)	97 (46)	54 (22)	218 (29.0)
30–49	169 (56)	74 (35)	141 (66)	384 (51.0)
> 50	62 (21)	23 (11)	22 (9)	107 (14.3)
Sex				
Female	186 (62)	132 (63)	121 (50)	439 (58.5)
Male	114 (38)	78 (37)	120 (50)	312 (41.5)
Education Level				
No Education	15 (5)	97 (46)	98 (41)	214(28.5)
Primary	235 (78)	84 (40)	105 (44)	420 (55.9)
Secondary	48 (16)	22 (11)	28 (12)	98 (13.1)
College	2 (7)	7 (3)	10 (4)	19 (2.5)
Occupation				
Businessmen	31 (10)	10 (5)	29 (12)	70 (9.3)
Pastoralist	17 (6)	150 (71)	59 (25)	227 (30.2)
Peasant	217 (72%)	20 (10)	57 (24)	294 (39.2)
Employed	15 (5)	19 (9)	15 (6)	49 (6.5)
Unspecified^a^	20 (7)	11 (5)	81 (34)	112(15)

^a^Those whose occupation was not specified during data collection

Out of 751 participants tested for anti-RVFV IgG, 34 tested positive making the overall sero-prevalence of 4.5% (95% C.I 3.2–6.3%) (Table 2). Of the 34 positive anti-RVFV IgG subjects, 6 (17.6%) tested positive for anti-RVFV IgM. Ngorongoro district recorded high seroprevalence of anti-RVFV antibodies of 8.1%, (17 of 210) compared to 2.1% recorded in Serengeti district (5 of 241) (*P* = 0.003) (Table 2). The seroprevalence of RVFV was significantly higher among pastoralists (8.9%, 20 of 227) compared to agro-pastoralists (3.4%, 10 of 294) (*p* = 0.008) (Table 2).

**Table 2:** Seroprevalence of anti-RVFV IgG by demographic factors in the Serengeti ecosystem, 2014

Variable	No. Enrolled	No. of IgG + VE	Seroprevalence (%)	*P* value
Total	751	34	4.5	
Age group				
< 15	42	0	0	0.96
30–49	384	16	4.2	0.61
> 50	107	7	6.6	0.57
15–29	218	11	4.4	Ref***
Sex				
Female	439	17	3.9	0.30
Male	312	17	5.5	
District				
Bunda	300	12	4.0	0.61
Ngorongoro	210	17	8.1	0.003
Serengeti	241	5	2.1	Ref
Level of education				
Informal	214	10	8.3	0.86
Primary	420	18	4.3	0.996
Secondary^b^	117	5	4.3	Ref
Occupation				
Businessman	70	4	5.7	0.36
Pastoralist	227	20	8.8	0.008
Others^a^	111	0	0	_
Employed	49	0	0	_
Peasant	294	10	3.4	Ref

^a^Those whose occupation was not specified during data collection

^b^Secondary include both with secondary and college education

***Ref- Reference group in Epi info

The odds of testing positive were almost 3 times higher among the pastoralists as compared to other occupational groups, which are small scale farmers, businessmen and employees. (aOR 2.9, 95% C.I: 1.21–6.89, *p* < 0.01) (Table 3). Those respondents who came from Ngorongoro (pastoral communities) were 2 times more likely to test positive for anti-RVFV IgG as compared to respondents from other districts (aOR 1.8, 95% C.I 1.14–3.39, *p* = 0.03) (Table 3). Age, sex and level of education were statistically not associated with exposure to RVFV (Table 3).

**Table 3:** Socio-demographic factors associated with RVFV sero-positivity in the Serengeti ecosystem, 2014

	Outcome			
Risk Factor	+ve	-ve	cOR (95% CI)	aOR (95% CI)
Occupation				
Pastoralist	20	205	3.56 (1.77–7.19	2.9 (1.21–6.89)
Others^a^	14	512	Ref*	Ref*
District				
Ngorongoro	17	193	2.71 (1.36–5.42)	1.8 (1.14–3.39)
Others	17	524	Ref*	Ref*
Education				
High education	5	93	1.15(0.44–3.06)	N/A
Low education	29	624	Ref*	
Age group				
< 29	11	249	0.91 (0.44–1.89)	N/A
30+	21	470	Ref	
Sex				
Male	17	295	1.43 (0.72–2.86)	1.5 (0.75–3.03)
Female	17	422	Ref*	Ref*

^a^All other occupational groups combined

Ref* - Reference group

The number of anti-RVFV IgG seroposivity was highest in Malambo, followed by Olbalbal, Nyerere (Serengeti) and Bunda health facilities. The seropositivity of IgM was determined from IgG seropositive samples only, and was sparsely distributed in Endulen, Piyaya, and Malambo in Ngorongoro district, as well as Bunda and Serengeti districts. Despite the higher anti-RVFV IgG seroprevalence in Olbalbal, no anti-RVFV IgM was detected (Fig. 1).

## Discussion

This study has shown past and recent exposure to RVFV in the Serengeti ecosystem, as both anti-RVFV IgG and IgM were detected. Anti-RVFV IgG antibodies are believed to last decades after infection and so provide a reliable index of prior RVF exposure. In contrast, anti-RVFV IgM has been reported to persist for only 6 to 8 weeks after initial infection [15]. Thus, finding anti-RVFV IgM antibodies does suggest recent exposure to the RVF infection among humans residing in the Serengeti ecosystem during interepidemic period. Living in Ngorongoro and being a pastoralist were the two common risk factors associated with exposure to RVF infection in our study.

The overall seroprevalence (4.5%) of RVF in the Serengeti ecosystem was slightly higher than (4%) previously reported in Tanga before the 2006/7 outbreak [17]. The slight increase of prevalence in this study might be because of cumulative exposure to RVFV infection in humans. Detection of anti-RVFV IgM among IgG seropositive humans was evidence that there is ongoing transmission of infection among humans during the interepidemic period. Lack of clinical RVF cases among anti-RVFV IgM positive humans might be due to the fact that the infections were sub-clinical or they were being missed or misdiagnosed for other disease conditions at health facilities, and this needs more studies. The ongoing transmission may be facilitated by the presence of several species of mosquitoes capable of spreading the disease [18, 19]. Some of these mosquitoes have been shown to be infected with RVFV in sister study to this (Nyarobi unpublished). In addition, eating raw animal products, including meat, blood and unpasteurised milk is a common practice among community members living in the study area [17, 20]. The detection might also be due to exposure to infected animals and wildlife in the area. The recorded anti-RVFV IgM seropositivity among anti-RVFV IgG reactive samples which were (17.6%) in this study, was low compared to that of (23%) recorded in study done shortly after 2006 outbreak [17]. However, there are several studies that didn’t find any anti-RVFV IgM seropositive individuals in spite of presence of anti-RVFV IgG seropositive in both human [18, 21] and other vertebrate samples [5]. A large sample size may be useful to detect presence of IgM against the disease during interepidemic period especially in humans.

The evidence of spatial distribution of cases in this study shows that Ngorongoro district had higher seroprevalence, especially in Malambo, Olbabal and Piyaya wards, all of which are typical pastoral communities. In Bunda and Serengeti, most of the seropositive individuals were found from people residing mostly where pastoral and agro-pastoral activities take place, while in Ngorongoro, cases were from typical pastoral communities. The findings is in line with previous studies, where number of clusters of RVF cases was found in several parts of Ngorongoro but neither in Bunda nor in Serengeti [8]. However, further research is needed to find out the reason behind this. It might be because of uncontrolled movement of live animals and animal products from Ngorongoro, although data on livestock movement is very scarce [8]. Some of the seropositive individuals in Bunda DDH were coming from the Lamadi ward in Busega district, which is bordering Bunda district, and near to where RVF virus nucleic acids was recently detected in competent vector mosquitoes by Nyarobi, (unpublished).

Geographically, Ngorongoro district had the highest seroprevalence of anti-RVFV IgG compared to Bunda and Serengeti districts. The large area of Ngorongoro district is sparsely populated and livestock keeping is the main activity of the residents, as no cultivation is allowed in the area. The higher exposure rate in Ngorongoro district could be attributed by the 2006/07 RVF outbreak [6]. High rainfall, high temperature and soil texture supportive of flooding in Malambo ward in Ngorongoro, and high animal density in the ecosystem may account for high disease prevalence in Ngorongoro district [8]. It is known that Rift Valley fever virus once introduced in the area, continues to exist for decades as it is maintained by Aedes mosquitoes through vertical transmission [22]. Thus effective surveillance should be enhanced in order to detect cases as early as possible as well as prediction of future outbreaks using satellite mapping. Likewise, community members should be informed on the current disease status, so that residents can be motivated to adhere with preventive measures in order to keep themselves safe from contracting the disease.

In this study, pastoralists were more exposed to RVFV with anti-RVFV IgG seroprevalence of 8.9% compared to other occupations 3.0%, which is in line with findings of other studies [23, 24]. This findings may be due to occupationally related risk of exposure exacerbated by high contact to infected animals and consumption of raw animal products such as blood, meat and milk [9]. Increased exposure might be enhanced because of poor community knowledge; attitude and practices related to RVFV infection and transmission pathways. For example, Maasai are used to keep their animals indoors during night to prevent them from wild carnivores’ attacks [20]. This practice increases the frequency of contact to animals and hence potential for animal-human transmission during outbreak.

The study was done in the Serengeti ecosystem, where several outbreaks of Rift Valley Fever have been reported previously [6, 7]. Thus the findings cannot be extrapolated to the entire country and may have limitation in generalization to other area of Tanzania. Likewise, as the study was cross-sectional in nature, it can only determine relationship at point in time and hence cannot determine temporal relationship. The study was designed as hospital-based study because of time limitation and financial constraints. The findings from this study can be used as an estimate of anti-RVF IgG and IgM seroprevalence in the Serengeti ecosystem, however, hospital-based study might have some bias and therefore it could be improved if it were supported by community survey.

Another potential limitation is not performing confirmatory virus neutralization test. However, comparison of IgG-sandwich and IgM-capture ELISA with virus neutralization test on field-collected sera from Africa (*n* = 2400) found the sensitivity of the IgG-sandwich ELISA was 100% and specificity 99.95%, while for the IgM-capture ELISA the values were 96.47 and 99.44%, respectively [19].

Following detection of anti-RVFV IgM, there is however, the need to conduct a study to determine the virulence of the viral strains circulating in the ecosystem. There might exist some strains, which are less virulent compared to the previous ones, or its virulence might have been altered by mutation due to lack of clinical cases during the study.

## Conclusion

The study found both previous and recent exposure of RVFV in humans residing in the Serengeti ecosystem as antibodies against both IgG and IgM were detected. Detection of anti-RVFV IgM suggests an ongoing transmission of RVFV in humans during interepidemic periods. Since recent exposure to the disease was found in this study, health care workers should consider RVF during their differential diagnosis of fever, especially for patients coming from pastoral communities, or those highly exposed to animals and raw animal products. Enhanced, well organized and effective surveillance system should be in place to detect cases as early as possible.

## Abbreviations

aOR: Adjust odds ratio
DDH: District designated hospital
GDP: Gross domestic product
GPS: Global positioning system
LGCA: Loliondo game controlled area
MUHAS: Muhimbili University of Health and Allied Sciences
NCA: Ngorongoro conservation area
OPD: Outpatient Department
OR: Odds ratio
RVF: Rift valley fever
TFELTP: Tanzanian field epidemiology and laboratory management training programme
